# Implementation of Transitional Care Innovations: Considering the Organizational Context and Process is Key

**DOI:** 10.1093/geroni/igab046.859

**Published:** 2021-12-17

**Authors:** Amal Fakha, Bram de Boer, Matheus van Achterberg, Jan Hamers, Hilde Verbeek

**Affiliations:** 1 Maastricht University, Maastricht, Limburg, Netherlands; 2 KU Leuven, Leuven, Vlaams-Brabant, Belgium

## Abstract

Many transitional care innovations (TCI) are implemented to improve long-term care services for older persons during the transition between various care settings. Nevertheless, multiple contextual factors (barriers; facilitators) influence the implementation of TCI at different levels such as but not limited to the organizational environment, outer setting, or innovation’s characteristics. By conducting a modified Delphi study involving 29 international experts from 10 countries, eleven influencing factors were prioritized and agreed upon (with ≥ 85% consensus level) as the most important for implementing TCI. These top factors were linked mostly to the organizational setting (e.g. resources, financing) or the implementation process (e.g. engaging key stakeholders). Moreover, the feasibility to address the majority of these factors with implementation strategies was rated as difficult. Our work concludes a compilation of major factors to be aware of and aim to tackle when preparing to implement a new TCI in any long-term care setting.

